# Medicinal Orchids of Mexico: A Review

**DOI:** 10.3390/ph17070907

**Published:** 2024-07-08

**Authors:** Luis J. Castillo-Pérez, Amauri Ponce-Hernández, Angel Josabad Alonso-Castro, Rodolfo Solano, Javier Fortanelli-Martínez, Luicita Lagunez-Rivera, Candy Carranza-Álvarez

**Affiliations:** 1 Programa Multidisciplinario de Posgrado en Ciencias Ambientales, Universidad Autónoma de San Luis Potosí, San Luis Potosí 78290, Mexico; jesus.perez@uaslp.mx; 2 Facultad de Estudios Profesionales Zona Huasteca, Universidad Autónoma de San Luis Potosí, Ciudad Valles 79060, Mexico; 3 Facultad de Ciencias Químicas, Universidad Autónoma de San Luis Potosí, San Luis Potosí 78290, Mexico; amauri.ponce@uaslp.mx; 4 Departamento de Farmacia, División de Ciencias Naturales y Exactas, Universidad de Guanajuato, Guanajuato 36050, Mexico; 5 Laboratorio de Extracción y Análisis de Productos Naturales Vegetales, Centro Interdisciplinario de Investigación para el Desarrollo Integral Regional, Unidad Oaxaca, Instituto Politécnico Nacional, Santa Cruz Xoxocotlán 71230, Mexico; asolanog@ipn.mx (R.S.); llagunez@ipn.mx (L.L.-R.); 6 Instituto de Investigación de Zonas Desérticas, Universidad Autónoma de San Luis Potosí, San Luis Potosí 78290, Mexico; fortanel@uaslp.mx

**Keywords:** Mexican orchids, traditional medicine, pharmacology, ethnomedicine

## Abstract

Some species of the Orchidaceae family are used in Mexican traditional medicine. However, there are no current and critical compilations of the medicinal uses and pharmacological effects of the members of the Orchidaceae family. This review provides a current, critical, and comprehensive analysis of the traditional medicinal uses, pharmacological reports, and active compounds isolated from Mexican orchids. A total of 62 Mexican orchids with medicinal potential have been recorded, of which 14 have scientific evidence. The remaining 48 plant species have ethnomedicinal information but have not been validated with scientific studies. These orchids are distributed in 14 states of the Mexican Republic, mainly in the southern region of Mexico. The most common pharmacological activities reported are anti-inflammatory, vasorelaxant, antinociceptive, antioxidant, spasmolytic, antihypertensive, and hallucinogenic activities. It is necessary to increase the number of pharmacological, phytochemical, and toxicological studies with medicinal orchids from Mexico because there are scientific studies on only 22.5% of these species. In further studies, it will be possible to evaluate the pharmacological effects of Mexican orchids in clinical trials. In addition, the mechanisms of action by which plant extracts and their active compounds exert medicinal effects remain to be studied. Plant extracts from orchids and their active compounds show promising antinociceptive and spasmolytic effects, respectively.

## 1. Introduction

Orchids are the most diverse group of flowering plants on the planet. Currently, more than 30,000 species are spread in a wide range of biogeographic regions. The distribution of the Orchidaceae family in the world is considered cosmopolitan because these plants are found in most of the planet’s ecosystems, except for the polar extremes and the most arid deserts [1].

Mexico has more than 1302 orchid species [2], which are most commonly used as ornaments due to the beautiful flowers that develop in different shapes and colors. However, several orchids in the country have apparent medicinal importance, and others, such as *Vanilla planifolia*, are used in the food industry [3]. Despite the great diversity of orchids that exist in the country, wild populations are decreasing due to anthropogenic activities, and, currently, 191 orchids are protected by the normative annex III of the Mexican normativity NOM-059-SEMARNAT-2010 [4], which includes the native flora and fauna species at risk.

Some Mexican orchids are used for medicinal purposes; however, few species have scientific research on the validation of their bioactive compounds or their pharmacological activities [3]. The validation of these properties is essential for obtaining drugs that can contribute to the prevention and treatment of several diseases [5]. Therefore, it is crucial to critically analyze the knowledge that is currently available on the ethnopharmacology of Mexican orchids and determine the gaps between traditional knowledge and evidence-based research.

To the best of our knowledge, there is not a current review that has been published on the aspects we discuss in this article about medicinal orchids from Mexico. Therefore, the main objective of this review is to provide updated and complete data on the distribution, ethnomedical uses, and phytochemical and pharmacology activities of the Mexican orchid species with medicinal potential. A bibliographic search was carried out for scientific reports in academic databases to analyze the diversity of medicinal orchids in Mexico. The information search was conducted in English and Spanish (due to the fact that Mexico is a Spanish-speaking country), and it was based on the following groups of keywords: Mexican medicinal orchids, Mexican orchid phytochemistry, and Mexican therapeutic orchids. The bibliographic search was conducted on the most relevant data in “PubMed”, “ScienceDirect”, “Scopus”, “Web of Science”, and “Google Scholar”, and in addition, physical and digital books were consulted. This review does not focus on *Vanilla planifolia* or its active compounds. Other reviews specifically address this orchid and can be consulted [6,7].

## 2. Distribution of Medicinal Orchids in Mexico

**Figure 1:**
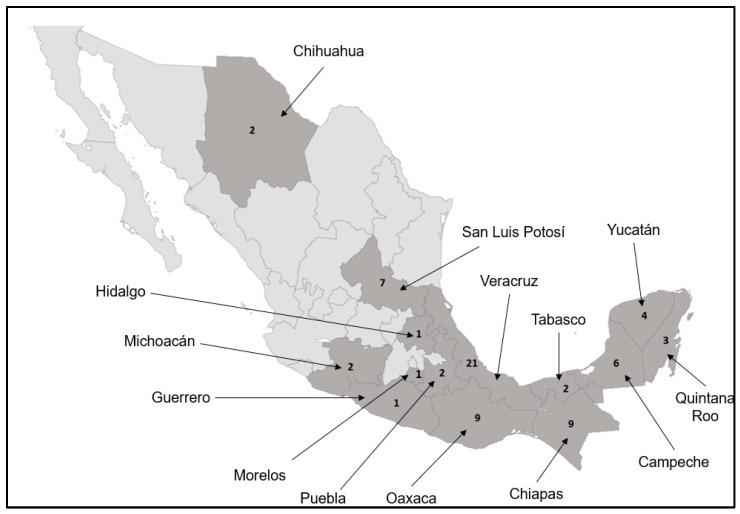
Number of orchids with medicinal potential used in every state in Mexico.

The distribution of the orchid species in Mexico is uneven. The humid forests in the tropical region of the country harbor the richest diversity of this family, whereas most of the northern part of the country presents environments unsuitable for these species [8]. Due to the marked distribution of the Orchidaceae family in the tropical southern region of Mexico, inhabitants from these states use orchids for medicinal purposes (Figure 1).

Table 1 shows 62 orchid species with medicinal properties used in Mexico. All the orchid names were corroborated and corrected, when necessary, by consulting the Missouri Botanical Garden (https://www.tropicos.org/home) and the International Plant Names Index (https://www.ipni.org) databases. Although all orchids are distributed in Mexico, not all are endemic to Mexico, and their distribution spreads to other countries on the American continent. These orchids have been reported in 14 of the 32 states of the Mexican Republic (Figure 1). Specific distributions for some species have not been reported, only the ethnic groups that use the plant. However, these data are inaccurate since many ethnic groups usually live in two or more states. For instance, Mayans live in several states in southern Mexico. For four orchid species, neither the state nor the ethnic group that uses the plants are registered (Table 1).

Veracruz was the state with the highest number of medicinal orchids (21 species), followed by the states of Chiapas (9 species), Oaxaca (9 species), San Luis Potosí (7 species), and Campeche (6 species) (Figure 1). Most of these territories have ecosystems such as cloud forests or rainforests, which are ideal for the growth and development of orchids [1,8], except for San Luis Potosí state, which has tropical weather and is in the central region of Mexico. This state is also the homeland of indigenous peoples, like the Tenek and Nahuatl, who use traditional medicine. The rest of the states are in the southern region of Mexico, where the tropical region is covered predominantly by jungles and rainforests. The characteristics of this region make it a suitable site for the growth of orchids and their use in ethnomedicine [64].

Chihuahua is the only state in northern Mexico with reports of medicinal orchids documented so far. In this state, two species have been reported, the Tepehuan ethnic group uses a species of the genus *Malaxis* for gastrointestinal diseases, and the Raramuri ethnic group uses *T. brachyphyllum* as a hallucinogenic during their religious ceremonies [59,60,61]. Mayans are another ethnic group that uses eight orchid species as medicinal plants, and the Nahuatl ethnic group uses seven orchid species to treat several ailments, especially those related to gastrointestinal diseases (Table 1).

In contrast, the medicinal properties of *Trichocentrum luridum* have been registered in seven states of the Mexican Republic, indicating that this orchid has a great range of distribution in Mexico (Table 1).

**Figure 2:**
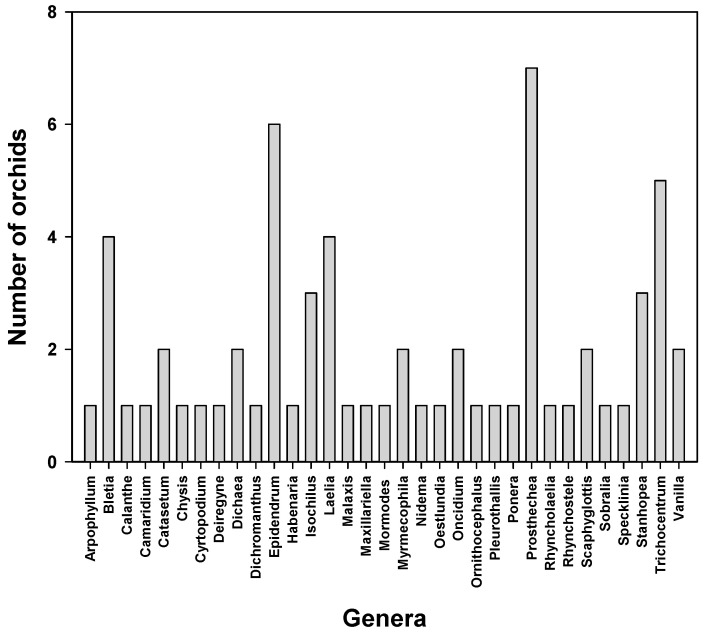
Genera of Mexican orchids with medicinal uses.

Regarding the diversity of medicinal orchids, 33 genera have been recorded. The genus *Prosthechea*, with seven species, presents the largest number of medicinal orchids in Mexico (Figure 2; Table 1), followed by the *Epidendrum* (six species), *Bletia* (four species), *Laelia* (four species), and *Trichocentrum* (three species) genera. Only two species of orchids are used for medicinal purposes in 18.2% of all the genera, whereas one orchid with medicinal purposes is reported in 60.6% of all the genera (Figure 2).

## 3. History of Ethnomedicinal Uses of Orchids

The wide herbal and cultural diversity in Mexico has resulted in a vast amount of ethnobotanical knowledge. Many indigenous communities living in rural areas of Mexico have preserved medicinal folk recipes, passed down from generation to generation [65]. These recipes have influenced the development of the current methodologies that seek to verify the validity of the therapeutic properties assigned to medicinal plants.

The Mexican population uses medicinal plants for the folk treatment of different diseases [5]. Of the 30,000 species of vascular plants in Mexico, between 3500 and 4000 have medicinal purposes. However, the chemical, pharmacological, and biomedical validation of the bioactive compounds has been carried out only in 5% of these species [66].

Regarding the Orchidaceae family in Mexico, the first reports of the medicinal use of Mexican orchids date back to the 16th century and correspond to the first herbal treaties based on native species of the country. Tlilxochitl (*Vanilla planifolia* Andrews) was the first native orchid of Mexico, the therapeutic uses of which were documented shortly after the arrival of the Spaniards. The medicinal uses of *V. planifolia* were reported in the *Libellus de medicinalibus indorum herbis*, a manuscript written in 1552 by Martin de la Cruz and translated into Latin by Juan Badiano [67], as well as in the *“Historia General de las Cosas de la Nueva España*” (*General History of the Things of New Spain*), written between 1540 and 1585 by Friar Bernardino de Sahagún [68]. Another manuscript from the early Mexican colonial period was written by Francisco Hernández between 1571 and 1577, describing and commenting on the medicinal uses of some orchids known to the Aztec people as tzauhtli, which means mucilage, a substance directly obtained from these plant species [10]. Furthermore, at the end of the colonial period, in 1801, Friar Juan Navarro wrote “*Natural History or American Garden*” [69] and documented the medicinal uses of *V. planifolia*.

In the second decade of the 19th century, Juan Lexarza described several orchids based on specimens collected in Michoacan. Some species were associated with the author Hernández [70]. In the early 20th century, Manuel Urbina determined the taxonomic names of Hernandez’s orchids, following Lexarza, and, in some cases, updated the names proposed by this author [9]. García-Peña and Peña [14] published the first review about the traditional uses of Mexican orchids, including medicinal species, and resorted to the names previously assigned by Urbina. Subsequent reviews on medicinal orchids [11,12,19,37,71,72,73,74,75], which included Mexican species, were mainly based on the names documented by the authors mentioned above.

Orchids with medicinal uses are more numerous in traditional medicines from China and India, and these countries stand out in the reviews on this subject. However, on the American continent, Mexico boasts the highest number of orchid species used for medicinal purposes, as reported by several authors [12,37,73]. Nevertheless, it is common in these studies for many of the names assigned to medicinal orchids from Mexico to have been based on Urbina [9], García-Peña and Peña [14], and Ospina [71], resulting in incorrect taxonomic identification. However, Hágsater et al. [1] have correctly identified the orchids documented by Hernandez, Lexarza, and Urbina.

## 4. Traditional Uses of Mexican Orchids

**Figure 3:**
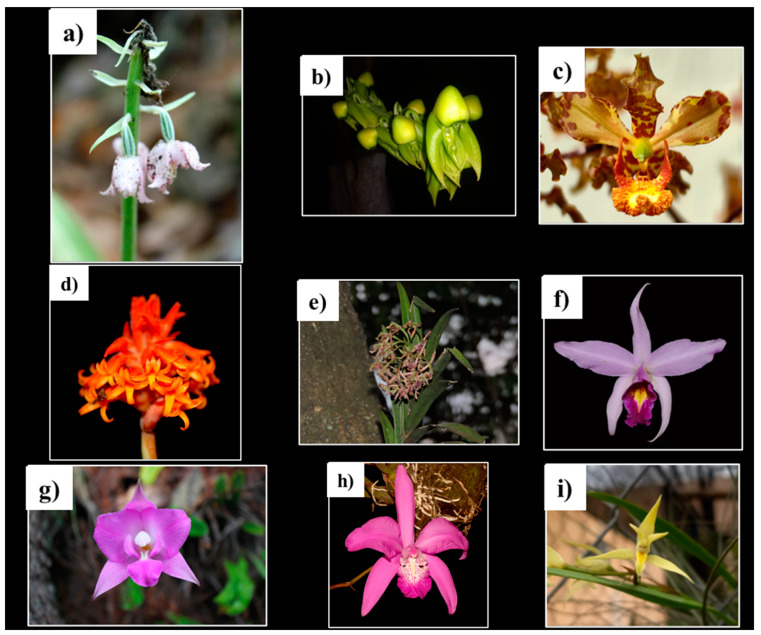

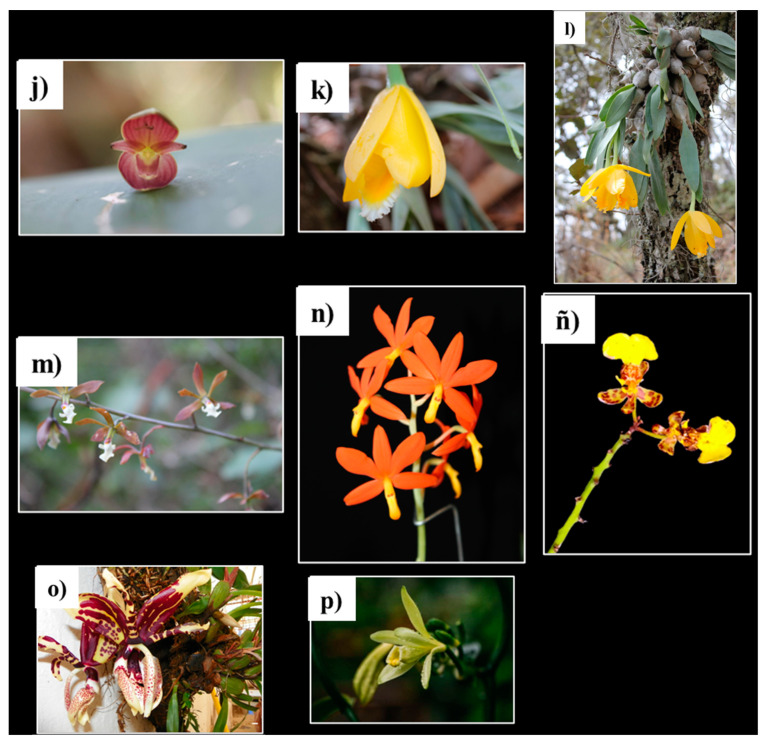
Mexican orchids with medicinal potential: (**a**) *Calanthe calanthoides* (A.Rich. & Galeotti) Hamer & Garay; (**b**) *Catasetum integerrimum* Hook.; (**c**) *Cyrtopodium macrobulbon* (Lex.) G.A.Romero & Carnevali; (**d**) *Dichromanthus cinnabarinus* (Lex.) Garay; (**e**) *Epidendrum anisatum* Lex.; (**f**) *Laelia anceps* Lindl.; (**g**) *Laelia furfuracea* Lindl.; (**h**) *Laelia speciosa* (Kunth) Schltr.; (**i**) *Nidema boothii* (Lindl.) Schltr.; (**j**) *Pleurothallis cardiothallis* Rchb.f.; (**k**) *Prosthechea citrina* (Lex.) Withner; (**l**) *Prosthechea karwinskii* (Mart.) Christenson; (**m**) *Prosthechea michuacana* (Lex.) W.E.Higgins; (**n**) *Prosthechea vitellina* (Lindl.) W.E.Higgins; (**ñ**) *Trichocentrum ascendens* (Lindl.) M.W.Chase & N.H.Williams; (**o**) *Stanhopea tigrina* Bateman ex Lindl.; (**p**) *Vanilla planifolia* Jacks. ex Andrews. Photographs by Rodolfo Solano, except (**b**) and (**i**) by Luis J. Castillo-Pérez, (**f**) by Candy Carranza-Álvarez, (**o**) by Javier Fortanelli and (**p**) by Alejandro Antonio-Calderon.

In this study, we found that of the 62 orchids recorded with medicinal uses, 77% are empirically used, but their biological activities have not been evaluated. Nevertheless, 14 species (22.5%) have reports on their pharmacological activities. Multidisciplinary work, including toxicological, pharmacological, and phytochemical investigations, are needed to evaluate the medicinal potential of the remaining 48 orchids without scientific evidence. Some of these orchids are shown in Figure 3. Orchids from the *Epidendrum* (six species), *Prosthechea* (six species), *Bletia* (four species), and *Laelia* (four species) genera have more orchids with medicinal purposes used in Mexican traditional medicine. Ethnomedicinal and pharmacological studies should be performed with other plant species from these genera.

In Mexican traditional medicine, 18 orchids (28.1%) (*Arpophyllum spicatum*; *Bletia reflexa*; *B. coccinea*; *B. campanulata*; *B. purpurea*; *Camaridium densum*; *Epidendrum anisatum*; *E. cnemidophorum*; *Isochilus lactibracteus*; *Isochilus* sp.; *Laelia autumnalis*; *Malaxis* sp.; *Maxillariella tenuifolia*; *Pleurothallis cardiothallis*; *P. pastoris*, *Prosthechea* sp. (probably *P. concolor*); *P. vitellina*; and *Vanilla planifolia*) are used to treat diseases related to the digestive system, such as dysentery and diarrhea, which are the main medicinal uses for orchids (Table 1).

The second most frequent use for medicinal orchids is pain and inflammation. Fifteen orchids are used for the folk treatment of pain and inflammatory diseases. These orchids are as follows: *Camaridium densum*; *Laelia anceps*; *Laelia speciosa*; *Mormodes maculata* var. *unicolor*; *Myrmecophila christinae*; *Myrmecophila tibicinis*; *Oestlundia luteorosea*; *Oncidium sphacelatum*; *Scaphyglottis fasciculata*; *Scaphyglottis livida*; *Sobralia macrantha*; *Stanhopea oculata*; *Trichocentrum ascendens*; *Trichocentrum brachyphyllum*; and *Vanilla planifolia* (Table 1). Gastrointestinal, respiratory, and musculoskeletal-related diseases are common among rural populations in Mexico, and medicinal plants are an alternative where no allopathic medicine is available for the general population [76]. In many rural areas, there is no access to potable water, which increases the possibility of gastrointestinal diseases. Musculoskeletal diseases, including rheumatoid arthritis, are common among the Mexican population. Approximately 1.5% of adults between 35 and 50 years old are diagnosed with rheumatoid arthritis [77].

It has been reported that *Catasetum integerrimum*, *Prosthechea karwinskii*, and *Prosthechea michuacana* have the potential to treat diabetes mellitus (Table 1), a disease that affects millions of Mexicans every year. In 2022, there were more than 14 million people with diabetes in Mexico, of which 4.5 million were not diagnosed [78].

The orchids *Dichaea muricata*, *Ponera striata*, *Specklinia* sp., *Trichocentrum brachyphyllum*, and *Vanilla mexicana* have been registered as medicinal, but the data for these species are scarce, with some studies only mentioning their uses and others only mentioning the places where these orchids are found and the ethnic groups that use them (Table 1). This indicates that more ethnobotanical studies carried out with Mexican orchids are needed. Our research group is focusing on providing more detailed information on the medicinal uses of medicinal orchids from Mexico and the methods of preparing and administering them.

Infusion is the most common way to prepare the medicine, and the main method of administration is by the oral route (Table 1). Some species of orchids are mixed with other plant species or some additive ingredients. For example, the leaves of *Dichromanthus cinnabarinus*, used to treat the common cold, are combined with *Monina* sp. leaves to form a kind of poultice [18]. The leaves and flowers of *Epidendrum radicans* are mixed with the leaves of *Brugmansia* sp. or with *Polygonum punctatum* to eliminate dandruff, pimples, coughs, and burns [18]. *Sobralia macrantha* is liquefied with water, lemon, and honey to decrease cough, fever, and inflammation [41,54]. Finally, the orchid *Stanhopea hernandezii* is mixed with red corn and other herbs to make tortillas, which are consumed to decrease weakness and a condition called “sunburn” [9,10] (Table 1). The pharmacodynamic and toxicological evaluations of these plant–plant combinations are topics for further research. Isobolographic studies might be helpful to evaluate the possible synergistic effects among combinations of medicinal plants.

## 5. Pharmacological Activity

**Figure 4:**
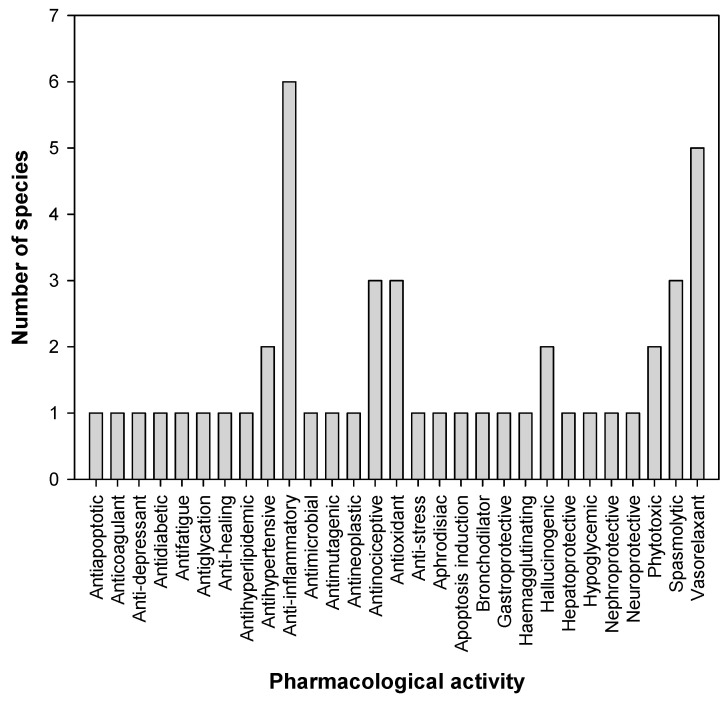
Frequency of medicinal orchids with pharmacological activities.

The pharmacological activities of 14 Mexican orchids have been reported using in vivo and in vitro models, which is equivalent to 22.5% of the 62 species used in Mexican traditional medicine. The most common pharmacological uses for Mexican orchids are anti-inflammatory uses (six species), followed by vasorelaxant (five species), antinociceptive, antioxidant, and spasmolytic uses (three species each) (Figure 4). A few studies have been conducted on animal models using mice or rats. In vitro studies should be corroborated with in vivo studies to validate the pharmacological evaluation. In addition, chronic studies might be considered before planning clinical trials.

The most studied species in terms of its pharmacological properties is *Vanilla planifolia*. However, most research focuses on the study and effects of vanillin (4-hydroxy-3-methoxybenzaldehyde), a molecule not exclusive to the species *V. planifolia* but also found in other species of the genus, such as *V. tahitensis* and *V. pompona* [79]. This molecule can also be obtained by chemical synthesis. Further pharmacological and chemical studies are necessary with other plant sections of *V. planifolia*, such as the stem, leaves, roots, or flowers. For the other species of Mexican orchids, the pharmacological research is described and analyzed in detail in the following sections.

### 5.1. Anti-Inflammatory Activity

Anti-inflammatory activity has been recorded for six species of Mexican orchids. This section discusses recent investigations into the anti-inflammatory activity of Mexican orchids. López-Pérez [38] tested a hydroethanolic extract (1000 μg/paw) of *Laelia furfuracea* leaves, an endemic orchid from the state of Oaxaca. This extract showed an inhibition of inflammation of 43.36% in a model of subplantar edema induced with carrageenan in Wistar rats. These results situate this orchid as a species that can be used to inhibit inflammatory processes, probably due to the presence of phenolic compounds.

A hydroalcoholic extract of the leaves (300 mg/kg p.o.) of the epiphytic orchid *Prosthechea karwinskii* showed an improvement in the gastroprotective effects by 70% and in the anti-inflammatory activity in a carrageenan-induced edema paw model with similar activity to that shown by naproxen (30 mg/kg). This extract showed the presence of compounds such as quinic acid, malic acid, neochlorogenic acid, chlorogenic acid, rutin, embeline, pinelic acid, and azelaic acid [45].

The anti-inflammatory activity of different extracts of *Prosthechea michuacana*, a terrestrial orchid, applied at 20 mg/mL, was determined using the model of 12-O-Tetradecanoylphorbol-13-acetate (TPA)-induced ear edema. The extracts that showed the highest anti-inflammatory activities were chloroform (leaves) (34.88%), hexane (rhizomes) (34.88%), hexane (pseudobulbs) (25.58%), and methanol (pseudobulbs) (16.27%) [46]. Another investigation conducted by Pérez-Gutiérrez and Vargas-Solís [47] found that the hexane extract from the leaves and pseudobulbs (200 mg/kg) of this orchid has significant anti-inflammatory activity before 3 h (74.02%), since it was proven to block the release of prostaglandins and cyclooxygenase in the later phase of the acute inflammatory process. Interestingly, the combined methanol–dichloromethane whole-plant extract of the orchid *Scaphyglottis livida* (150–600 mg/kg) reduced the inflammatory process induced by carrageenan. These data suggest that this orchid has a significant dose-dependent, anti-inflammatory effect in rats and mice [17].

In this review, we report three investigations that adjudged the anti-inflammatory potential of the vanillin molecule, one of the natural and major components of *Vanilla planifolia*. Eun-Ju et al. [80] performed vascular permeability tests and observed that the vanillin showed inhibitions of the inflammatory process of 14.2%, 40.6%, and 49.8% at concentrations of 25, 50, and 100 mg/kg, respectively. For their part, Kwon et al. [81] developed a new inflammation-responsive polymeric vanillin antioxidant prodrug called poly vanillin oxalate (PVO), which achieved potent anti-inflammatory activity by reducing the expressions of proinflammatory cytokines in activated macrophages in vitro and in vivo. Finally, Niazi et al. [82] determined that with the administration of doses of 50 and 100 mg/kg of vanillin, a significant decrease in the inflammation observed in the volumes of the mouse paws was achieved, which they related to its antihistamine activity.

The evidence shown in these investigations suggests that the ingredients of these orchids might be used to create a safe drug that increases the anti-inflammatory potential. Chronic studies are necessary to validate the efficacy shown by orchids in acute-term studies. Several models of chronic inflammation, including TPA-induced ear edema, kaolin–carrageenan-induced monoarthritis, Freund’s complete adjuvant (FCA)-induced arthritis, and others, could be used with Mexican orchids. The effect of Mexican orchids on molecular targets of inflammation, such as cyclooxygenase-2 (COX-2), nuclear factor κB (NF-κB), tumor necrosis factor-alpha (TNF-α), and others, could be evaluated.

### 5.2. Vasorelaxant Activity

The vasorelaxant activity has been assessed in five orchid species, of which three belong to the *Laelia* genus. Vergara-Galicia et al. [31] made a methanolic extract with *Laelia anceps* roots, and when administered at 50 µg/mL, a relaxation of 64.37% was presented in the aortic rings with the endothelium. The vasorelaxant effect shown by the extract was not inhibited by the co-administration with 1H-[1,2,4]Oxadiazolo [4,3-a]quinoxalin-1-one (ODQ), 1-alprenolol, glibenclamide, or 2-Aminopurine (2-AP). This effect was associated with the presence of the compound 2,7-dihydroxy-3,4,9-trimethoxyphenanthrene, characterized by a High-Performance Liquid Chromatography (HPLC) analysis. Vergara-Galicia et al. [32] made two extracts with *Laelia anceps*, one with hexane and another with dichloromethane, and although the pseudobulbs and leaves were used, the root extracts were the most active (effective concentration 50, EC_50_ = 6.78 µg/mL with the endothelium and EC_50_ = 6.78 µg/mL without the endothelium).

Aguirre-Crespo et al. [35] prepared a methanolic extract with the aerial parts of *Laelia autumnalis* and observed the induction of a relaxation process (inhibitory concentration 50, IC_50_ = 4.37 × 10^−8^ ± 1.77 × 10^−8^ M) dependent on the concentration and independent of the endothelia in the aortas of rats. L-NG-Nitro arginine methyl ester (L-NAME) blocked relaxation, indicating that the vasodilatory properties of the extract are mediated by the endothelium due to the release of nitric oxide. Moreover, Vergara-Galicia et al. [36] obtained a methanolic extract using roots, pseudobulbs, leaves, and seedlings generated in vitro. This is one of the few works in which orchid vitroplants were used to investigate the vasorelaxant action. However, the roots (maximum effect, Emax = 64.37 ± 3.43% at 50 µg/mL with endothelium-intact aorta rings) and pseudobulbs (Emax = 83.32 ± 3.55% at 50 µg/mL with endothelium-intact aorta rings) of *L. autumnalis* were the extracts that produced the best vasorelaxant effects in a concentration-dependent and endothelium-independent manner on contractions induced by norepinephrine (NE) and potassium chloride (KCl) in rat thoracic aorta rings. Leaf and in vitro seedling extracts did not show relevant vasorelaxant activities. Finally, Aguirre-Crespo et al. [35] observed that *L. autumnalis* induced relaxation (IC_50_ = 34.61 ± 1.41 µg/mL and Emax = 85.0 ± 4.38% in endothelium-intact aorta rings) and determined that the extract of this orchid caused an inhibition of the sustained contraction of serotonin. The vasorelaxant effect of rat aortic rings is carried out through an endothelium-independent pathway, which implies the blockade of Ca^2+^ channels and a possible enhanced concentration of cyclic guanosine monophosphate (cGMP).

*Laelia speciosa* is the third species of the *Laelia* genus for which the vasorelaxant activity was investigated, specifically with hexane, dichloromethane, and methanol extracts from the roots and pseudobulbs. The results showed that all the extracts tested induced a concentration-dependent relaxation process in the precontracted aortic rings with and without the endothelium. However, the dichloromethane and hexane root extracts were less potent than the positive controls used (carbachol and sodium nitroprusside). A methanol extract of *Laelia speciosa* roots (EC_50_ = 2.75 µg/mL in aorta rings with the endothelium) showed the highest vasorelaxant activity. These results suggest that the secondary metabolites responsible for the vasorelaxant activity belong to a group of compounds with medium and low polarities, and that the roots are the main tissues of the plant where the vasorelaxant compounds are stored [32].

In the case of the orchid *Scaphyglottis livida*, the stilbenoid compounds gigantol (IC_50_ = 1.68 × 10^−6^ ± 0.052 M in endothelium-intact aorta rings) and 3,7-dihydroxy-2,4-dimethoxyphenantrene (IC_50_ = 5.10 × 10^−5^ ± 0.55 M in endothelium-intact aorta rings) were found to induce a significant concentration-dependent relaxation of norepinephrine-evoked contractions in endothelial-bearing rat aortic rings intact and naked [53].

Through the use of ex vivo assays, these works indicate that orchids have vasorelaxant actions, and that they should continue to be investigated from this perspective because these plant species could be a source of antihypertensive drugs. Chronic-term studies using in vivo models should also be considered to evaluate the antihypertensive actions of Mexican orchids.

### 5.3. Antinociceptive Activity

Research on antinociceptive activity is important to find biomolecules in plant species that can be used for pain treatment. This biological activity has been verified in Mexican medicinal orchids. Firstly, a combined dichloromethane–methanol extract of *Camaridium densum* (150–600 mg/kg p.o.), an orchid formerly known as *Maxillaria densa*, reduced acetic acid-induced abdominal writhing but failed to produce antinociception in the hot-plate test. In addition, the researchers were able to isolate two chemical compounds from this orchid, fimbriol A (Emax = 69% at 25 mg/kg p.o.) and erianthridin (Emax = 65% at 25 mg/kg p.o.), which partially reduced the contortions induced by acetic acid. This study showed that the extract used is relatively non-toxic and should be regarded as safe. Moreover, the doses tested did not provoke any side effects [17].

A second orchid species with antinociceptive potential is *Cyrtopodium macrobulbon*. Two research teams in Mexico have studied this orchid, specifically the pseudobulbs. Morales-Sánchez et al. [23] analyzed an aqueous extract and an organic extract with contortions induced by the acetic acid methodology and the hot-plate test and observed that only the organic extract of the pseudobulbs showed an improvement in the antinociceptive effect by 30% when administered at doses of 100 mg/kg.

Yáñez-Barrientos et al. [83] analyzed the roots of *Laelia anceps* and *Cyrtopodium macrobulbon*. Ethanolic extracts of *C. macrobulbon* (effective dose 50, ED_50_ = 17.8 mg/kg) and *L. anceps* (ED_50_ = 48.4 mg/kg) showed antinociceptive activity in the acetic acid-induced-writhing test. *C. macrobulbon* (ED_50_ =198 mg/kg in phase 1 and ED_50_ = 29 mg/kg in phase 2) and *Laelia anceps* (ED_50_ = 186 mg/kg in phase 1 and ED_50_ = 97 mg/kg in phase 2) also showed antinociceptive activity in the formalin test. Pre-treatment with L-NAME reverted the antinociceptive effects of the *L. anceps* extract and *C. macrobulbon* extract in the acetic acid test.

At this point of the review, it is important to mention that some assessments of the bioactivities of certain Mexican orchids have been conducted using extracts obtained from the roots of *Cyrtopodium macrobulbon* [83], *Laelia anceps* [31,32,83], *L. autumnalis* [36], *L. speciosa* [32], *P. michuacana* [46], *Stanhopea tigrine* [84], and *Trichocentrum Brachyphyllum* [85]. However, adult orchid plants still maintain associations with fungi, which form their mycorrhizae, with varying degrees of colonization [86]. Since most orchid roots consist of non-living cells and fungi that produce compounds with significant biological activities, it is unclear whether the effects reported by these studies are due to secondary metabolites produced by the orchid or its fungal endophytes. Studies are needed to verify which organism is responsible for the reported activity.

Finally, for the orchid *Scaphyglottis livida*, Déciga-Campos et al. [17] used the contortion test and analyzed the antinociceptive effects of a methanolic extract and another extract made with dichloromethane using the whole plant. The results showed that doses of 150, 300, and 600 mg/kg of the orchid methanolic extract reduced the number of stretches in the acetic acid-induced contortion test by 40–50%. Subsequently, the antinociceptive effects of four compounds isolated from *S. livida* were analyzed using the hot-plate test. The compounds 5α-lanosta-24,24-dimethyl-9(11),25-dien-3β-ol, and gigantol significantly increased the hot-plate latency compared to the vehicle-treated mice [17].

The antinociceptive activity of Mexican orchids seems promising, since *Cyrtopodium macrobulbom* has shown activities similar to those of reference drugs. In addition, the antinociceptive activities of several active compounds from Mexican orchids have been evaluated. The molecular mechanisms, including the inhibition of COX-2 and other molecules involved in the pain pathway, remain to be studied, especially with the active compounds.

### 5.4. Antioxidant Activity

Three Mexican medicinal orchids have antioxidant activity, two of which belong to the genus *Prosthechea*. In the case of the orchid *P. karwinskii*, the antioxidant capacity was determined by the 2 2-diphenyl-1-picrylhydrazyl (DPPH) method, and with these data, the IC_50_ and the antioxidant activity index (AAI) were generated. The highest antioxidant activity index was found in the leaf extract (AAI = 5.7), followed by the flower extract (AAI = 1.276) and pseudobulb extract (AAI = 0.925) [43].

Pérez-Gutiérrez et al. [50] used *P. michuacana* pseudobulbs and determined the antioxidant activity through the total content of phenols, the DPPH radical-scavenging effect, and the 2 2′-azino-bis(3-ethylbenzothiazoline-6- sulfonic acid) (ABTS) radical-scavenging test. This study reported the extraction and isolation of six compounds: two triterpenoids (3α-acetoxy, 24-hydroxy-24-methyl-5α-lanosta-9(11),25-diene (1) and 3α-acetoxy, 24- hydroxy -24-methyl-5α-lanosta-9(11)ene (2)); one stilbene (α-α′-dihydro,3′,5′,2-trimethoxy-3-hydroxy-4-acetyl-4′-isopentenylstilbene (3)); one phenanthrene (4,6,7-trihydroxy-2-methoxy-8-(methylbut-2-enyl)phenanthrene-1,1′-4′,6′,7′-trihydroxy-2′-methoxy-8′-(methylbut-2′-enyl) phenanthrene (4)); one diterpene (12-hydroxy-3β,7β,18α-triacetoxy-8,11,13-abietatriene (5)); and gigantol (6). Of these compounds isolated from *P. michuacana*, 4 (IC_50_ = 7.5 µg/mL in DPPH assay) and 5 (IC_50_ = 15.4 µg/mL in DPPH assay) showed the best antioxidant capacities.

The third orchid with studies on its antioxidant properties is *Vanilla planifolia*. Shyamala et al. [87] used vanilla fruits extracted with 60% aqueous ethyl alcohol (IC_50_ = 385 mg/L) and identified and purified the compounds vanillic acid, 4-hydroxybenzyl alcohol (IC_50_ = 220 mg/L), 4-hydroxy-3-methoxybenzyl alcohol (IC_50_ = 154 mg/L), 4-hydroxybenzaldehyde, and vanillin. Vanillic acid and vanillin did not cause 50% inhibition in the β-carotene linoleate method.

Some compounds (4 and 5) found in the study of Pérez-Gutiérrez et al. [50] showed similar antioxidant effects to those of reference compounds. Further, in vivo studies should be carried out with these compounds. The antioxidant activity of natural products, like these two compounds, can be used in the prevention and development of several chronic diseases. Protocols for the synthetic synthesis of compounds 4 and 5 are highly desirable. In addition, using biotechnological approaches can be useful to produce these two compounds.

### 5.5. Spasmolytic Activity

Three orchids used to reduce pain caused by contractures, spasms, and injuries have been recorded. Two compounds, derived from phenanthrenes, obtained from a *Camaridium densum* whole plant extracted with dichloromethane–methanol (IC_50_ = 0.62 ± 0.13 µg/mL), and their active compounds, 2,5-Dihydroxy-3,4-dimethoxyphenanthrene (IC_50_ = 0.95 µM), fimbriol-A (IC_50_ = 0.67 µM), nudol (IC_50_ = 0.73 µM), gymnopusin (IC_50_ = 5.8 µM), and erianthridin (IC_50_ = 7.1 µM), showed smooth-muscle-relaxant properties in the rat ileum. These compounds showed similar or higher potencies to that of papaverine (IC_50_ = 7.1 µM) [16]. The nitrergic and histaminergic systems, as well as the interference with the calcium influx in the smooth-muscle cells, are the possible mechanisms of these compounds [16].

A dichloromethane–methanol extract with the whole plant of *Nidema boothii* inhibited (IC_50_ = 6.26 ± 2.5 µg/mL) guinea-pig ileum contractions. Several compounds, including batatasin III (IC_50_ = 0.24 ± 0.11 µM), gigantol (IC_50_ = 0.26 ± 0.10 µM), lusianthridin (IC_50_ = 0.41 ± 0.03 µM), and 1,5,7-trimethoxyphenanthrene-2,6-diol (IC_50_ = 0.45 ± 0.03 µM) were isolated from the plant extract through bioassay-guided fractionation, and their spasmolytic activity was recorded [88].

The five aromatic compounds isolated from *Scaphyglottis livida*, 3,4′-dihydroxy-5,5′-dimethoxybibenzyl (IC_50_ = 5.8 µM), batatasin III (IC_50_ = 0.73 µM), coelonin (IC_50_ = 0.95 µM), 3,7-dihydroxy-2,4-dimethoxyphenanthrene (IC_50_ = 0.67 µM), and 3,7-dihydroxy-2,4,8-trimethoxyphenanthrene (IC_50_ = 7.1 µM), inhibited the contraction in the rat ileum, and the nitric oxide/cGMP pathway seems to be involved in the spasmolytic activities of these compounds [15].

**Figure 5:**
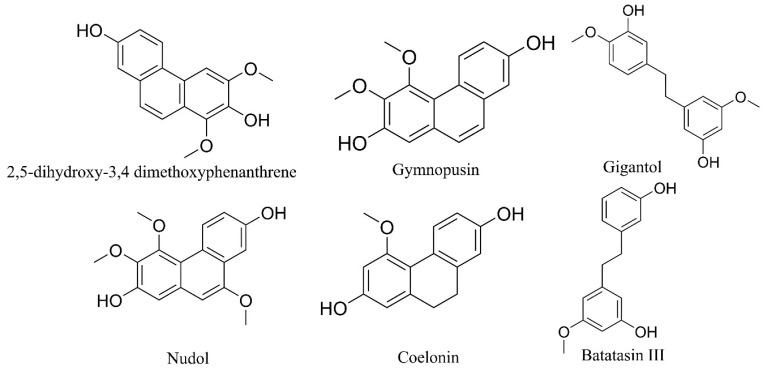
Chemical structures of compounds with promising spasmolytic activities.

The molecular mechanism of the spasmolytic effects exerted by nudol and gymnopusin, isolated from *Camaridium densum*, gigantol, and batatasin III, obtained from *Nidema boothii*, and 3,7-dihydroxy-2,4-dimethoxyphenanthrene and coelonin, obtained from *Scaphyglottis livida*, should be evaluated (Figure 5). As mentioned above, these compounds showed higher activities compared to the reference drugs. More molecular studies, in vivo assays, and toxicological studies are required with these promising compounds with spasmolytic activities.

### 5.6. Antihypertensive Activity

In Mexico, there are two orchids of the *Laelia* genus for which effective antihypertensive activity has been proven. A methanolic extract of *Laelia autumnalis* (100 mg/kg p.o.) decreased the systolic and diastolic blood pressures by 20%, as well as the heart rates, of spontaneously hypertensive rats after 2 h of treatment. Furthermore, the antihypertensive effect shown by the plant extract is caused by the blockade of Ca^2+^ channels and a possible enhanced concentration of cGMP [35].

A methanolic extract of *Laelia anceps* roots (100 mg/kg p.o.) decreased the systolic and diastolic blood pressure by 25% in hypertensive rats after 2 h of treatment. The reduction in the transient contraction induced by norepinephrine in a solution free of Ca^2+^ ions and the inhibition induced by the increase in external calcium suggest that the antihypertensive effect caused by the plant extract is conducted by blocking the channels of Ca^2+^ [31].

Arterial hypertension has had negative impacts on health in many countries. This condition represents a predisposition for the development of diseases such as metabolic syndrome, diabetes, kidney dysfunction, heart failure, and stroke [89]. Therefore, the development of research for the discovery of drugs that contribute to the treatment of these diseases is urgent. The results that the species of the *Laelia* genus have provided in terms of the discovery of metabolites with hypertensive activity suggest that this genus of orchids could be an excellent source of these compounds. Interestingly, in Mexico, there are 11 species belonging to this genus, of which, as shown in this research, only 2 have been studied.

### 5.7. Other Pharmacological Activities

Other pharmacological activities have been conferred on Mexican medicinal orchids. For example, Zenteno et al. [34] reported that, in *Laelia autumnalis*, the pseudobulbs have hemagglutinating properties four-fold higher in the presence of human A1 erythrocytes. Barragán-Zarate et al. [44] verified the anticoagulant activity of hydroalcoholic extracts of *Prosthechea karwinskii* pseudobulbs, leaves, and flowers. The three extracts prolonged the activated Partial Thromboplastin Time (aPTT) by approximately 15–20%. A hydroalcoholic extract of *Prosthechea karwinskii* leaves (100–1000 mg/kg p.o.) showed gastroprotective effects in a model of indomethacin-induced gastric ulcers in rats after 3 h of treatment [45].

After 6 h of treatment, hexane extracts of *Prosthechea michuacana* bulbs (400 mg/kg p.o.) decreased the levels of blood glucose (47.78%), cholesterol (46.08%), and triglycerides (46.27%) in streptozotocin-induced diabetic rats [52]. This extract (IC_50_ = 74.6 µg/mL) and the active compounds α-α′-dihydro, 3′,5′,2-trimethoxy-3-hydroxy-4-acetyl-4′-isopentenylstilbene (IC_50_ = 24.5 µM), 4,6,7-trihydroxy-2-methoxy-8-(methylbut-2-enylphenanthren)-1-1′-4′,6′,7′-trihydroxy-2′-methoxy′-(methylbut-2′-enyl) phenanthrene (IC_50_ = 71.6 µM), and gigantol (IC_50_ = 28.7 µM) also decreased the formation of advanced glycation end products (AGEs) using bovine serum albumin glycated in the presence of glucose [52].

The other biological activities that have been verified in the bulbs of this Mexican medicinal orchid are hepatoprotective and nephroprotective activities. These data situate *P. michuacana* as an important orchid for the manufacture of possible drugs for the treatment of diabetes mellitus, one of the diseases that currently afflicts Mexico and the world the most.

Ethanol extracts of *Stanhopea tigrina* leaves showed anxiolytic-like activities in the exploratory-cylinder test (ED_50_ = 49.34 mg/kg p.o.) and the hole-board test (ED_50_ = 11.52 mg/kg p.o.) without producing hypnotic, sedative, or locomotor impairments. The mechanisms of the anxiolytic-like effects of this plant extract are due to the involvement of the GABAergic, adrenergic, and serotonergic systems. At 50 and 100 mg/kg p.o., this plant extract exerted diuretic effects with the excretion of sodium and, to a lesser extent, potassium. The possible mechanism of the diuretic action of *Stanhopea tigrina* is through the participation of nitric oxide [56].

For *Vanilla planifolia*, it is worth discussing the biological activities of this orchid separately because what has been studied is mainly the vanilla molecule and very rarely the extract of some section of the plant. Therefore, we could consider the vanillin molecule as an excellent medicinal phytochemical compound, but not the *Vanilla planifolia* plant, because, in many cases, the molecule is synthesized in the laboratory and is not extracted naturally.

## 6. Active Compounds

The bioactive compounds isolated from the Orchidaceae family began to be studied approximately 25 years ago, and despite the great variety of species that this botanical family possesses, there are scarce reports about its phytoconstituents [44].

Analyses on the chemical composition, isolation, and identification of the compounds in the different parts of orchids (roots, leaves, pseudobulbs, stems, and flowers) have been performed by research groups from around the world [90].

This review presents the main compounds (terpenes and phenolic compounds) found in medicinal orchids used in Mexican traditional medicine. In the case of alkaloids, there is a study reporting the presence of alkaloids in *Trichocentrum cebolleta*, reported to have hallucinogenic activity [60]. In this review, 13 orchid species of the total 62 orchids reported with medicinal use in Mexico have studies on their chemical compositions.

### 6.1. Terpenes

Terpenes are the largest group of plant secondary metabolites, with more than 55,000 known chemical structures [91]. Terpenes are generally insoluble in water and are formed by the union of five-carbon compounds called an isoprene. Some terpenes produced by orchids and other plants attract insects, promote pollination, and produce toxins and repellents to defend plants from attacks by insects, herbivores, or pathogens, or their synthesis is due to a response to abiotic stress [92]. Some terpenes have shown antibiotic, antifungal, antiviral, and anticancer activities [91,93].

The presence of terpenes with biological importance has been reported for seven species of Mexican orchids. In the aerial parts of *Cyrtopodium macrobulbon*, three volatile terpenes, namely, 6,10,14-trimethyl-2-pentadecanone, eucalyptol, and isobornyl formate, have been reported. Among these terpenes, only eucalyptol showed antinociceptive activity [23]. Two triterpenes with antiasthma activity (24,24-dimethyl-9,19-cyclolanostane-25-en-3β-ol and 24-methyl-9,19-cyclolanost-25-en-3β-ol) were isolated from *Epidendrum rigidum* [94].

López-Pérez [38] reported the presence of sesquiterpene lactones, saponins, and cardiotonic glycosides in *Laeila furfuracea*. Among these molecules, sesquiterpene lactones and saponins have been identified as potent antioxidants, whereas cardiotonic glycosides produce effects on the heart muscle, specifically on Na^+^/K^+^ ATPases [95]. Triterpenes were reported in *Prosthechea karwinskii* and *Stanhopea hernandezii*. However, these molecules were not characterized. Similarly, the presence of four triterpenes was found in *Scaphyglottis livida*, and 5α-lanosta-24,24-dimethyl-9(11),25-dien-3β-ol (LDD), 5α-lanosta-24(S)-methyl-9(11),25-dien-3β-ol, 24,24-dimethyl-9,19-cyclolanosta-9(11),25-dien-3-one (cyclobalanone), and 9,19-cyclolanosta-24,24-dimethyl-25-en-3β-yl trans-p-hydroxycinnamate were identified.

Two triterpenes were isolated from the chloroform extract of *P. michuacana* bulbs, 3α-acetoxy,24-hydroxy-24-methyl-5α-lanosta-9(11),25-diene and 3α-acetoxy,24-hydroxy-24-methyl-5α-lanosta-9(11)ene, and a diterpene was identified as 12-hydroxy-3β,7β,18α-triacetoxy-8,11,13-abietatriene. According to the results obtained in the DPPH radical-scavenging tests, this diterpene showed antioxidant activity [50].

The presence of terpenoids in hexane and methanol/ethanol extracts from *Vanilla planifolia* leaves and stems has been reported in the literature, but the studies are just qualitative methodologies [96]. It is necessary to start with the characterization of these extracts and to identify the types of terpenes. However, chemical characterizations of some extracts for the identification of the compounds they contain have not been carried out, so this could be an important field of study that will add information on orchid terpenes.

### 6.2. Phenolic Compounds

Phenolic compounds, mainly of plant origin, are secondary metabolites with an aromatic ring and hydroxyl substituents. Some of these compounds act as a defense against herbivores and pathogens, such as via mechanical support, as pollinator attractors, and through plant allelopathy processes [97]. Stilbenoids and phenanthrenes are phenolic compounds found in the Orchidaceae family [98]. Orchid phenanthrenes have shown pharmacological activities, such as antimicrobial and antiproliferative effects against cancer cells and anti-inflammatory and antioxidant effects. The findings in Mexican medicinal orchids related to phenolic compounds are detailed below.

Phenanthrene derivatives like 9,10-dihydro-2,5-dihydroxy-3,4-dimethoxyphenanthrene, 2,5-dihydroxy-3,4-dimethoxyphenanthrene, nudol, erianthridin, fimbriol-A, and gymnopusin have been isolated and identified in *Camaridium densum*.

In *Cyrtopodium macrobulbon*, 10 different phenolic compounds were isolated from an organic extract of the pseudobulbs. Among these phenolic compounds, there are three derivatives of p-coumaric acid (n-hexacosyl-trans-p-coumarate, n-octacosyl-trans-p-coumarate, and n-triacontyl-trans-p-coumarate). Studies have indicated that p-coumaric acid and its derivatives can protect mammalian cells in culture against oxidative stress and genotoxicity [99]. Additionally, p-coumaric acids have shown antioxidant and cytotoxic effects [100]. The rest of the phenolic compounds isolated from *C. macrobulbon* are the stilbenoids 4-methoxy-benzylalcohol, 4-hydroxybenzaldehyde, 1,5,7-trimethoxy-9,10-dihydrophenanthrene-2,6-diol, confusarin, gigantol, batatasin III, and ephemeranthol B [23]. Of these compounds, gigantol and ephemeratrol B are known to possess antinociceptive, anti-inflammatory, and antioxidant activities.

One flavonol and two phenanthrenes (9,10-dihydro-2,5-dimethoxyphenanthrene-1,7-diol and 2,7-dihydroxy-3,4,9-trimethoxyphenantrene) were reported in the epiphytic orchid *Laelia anceps*. These phenanthrene-type compounds have an anti-inflammatory effect, whereas 5-hydroxy-3,7,4′-trimethoxyflavonol has antimutagenic, antifungal, antibacterial, and cytotoxic activities [30].

The presence of 13 phenolic compounds was recorded in *Laelia furfuracea*, used in the prevention of cardiovascular diseases. Syringic acid acetate, one of these compounds detected in the hydroethanolic extract of the leaves, reduced the coagulation times and had antioxidant, anti-inflammatory, and neuro- and hepatoprotective activities [101]. Protocatechuic acid possesses antithrombotic and antiplatelet activities, inhibits the granular secretion of dense granules and α-granules, and attenuates the activation of glycoproteins IIb/IIIa [102]. Rosmarinic acid has antioxidant, platelet inhibition, and anticoagulant activities [103]. Luteolin-7,3′-di-O-glucoside has antiplatelet and vasorelaxant activities, and kaempferol-3-O-rutinoside and kaempferol-7-O-glucoside have anticoagulant activity. The stilbenoid 4-hydroxybenzyl alcohol (HBA) has shown antiangiogenic, anti-inflammatory, and antinociceptive activities. Finally, malic acid and caffeic acid have antioxidant, anti-inflammatory, and cytotoxic effects [38].

The orchid *Nidema boothii* is not commonly used in traditional medicine. However, scientific evidence has shown that this orchid has a variety of phenolic compounds, including stilbenoids and phenanthrene derivatives. According to Hernández-Romero et al. [88], nine phenolic compounds have been found in this orchid, and, among them, six are derivatives of phenanthrenes (1,5,7-trimethoxyphenanthrene-2,6-diol; 1,5,7-trimethoxy-9,10-dihydrophenanthrene-2,6-diol; 2,4-dimethoxyphenanthrene-3,7-diol; ephemeranthol B; ephemeranthoquinone; and lusianthridin) and three are stilbenoids (aloifol II, batatasin III, and gigantol). The compounds aloifol II, 1,5,7-trimethoxy-9,10-dihydrophenanthrene2,6-diol, 1,5,7-trimethoxyphenanthrene2,6-diol, ephemeranthoquinone, gigantol, 2,4-dimethoxyphenanthrene-3,7-diol, lusianthridin, and batatasin III have concentration-dependent spasmolytic activity in mouse ilea [15].

Phenolic constituents such as 7-glucoside, caffeic acid, tyrosol, apigenin, vanillin, p-coumaric acid, and ferulic acid were identified in *Prosthechea karwinskii*. These compounds are known for their cardioprotective activity due to their ability to inhibit cholesterol oxidation [43], and some of these phenols inhibit the growth of adipose tissue due to their antiangiogenic activity [104].

Caffeoyl-quinic acids (chlorogenic acid and neochlorogenic acid) and flavonoid glycosides (rutin and kaempferol-3-O-rutinoside) were isolated from *Prosthechea karwinskii* [45]. These flavonoid glycosides showed anti-inflammatory and antidiabetic activities due to the inhibition of the mitogen-activated protein kinase (MAP kinases) and the kappa-light chain of the NF-κβ signaling pathways [105,106]). Rutin, kaempferol-3-O-rutinoside, and chlorogenic acid decreased the expression of COX-2. Also, chlorogenic acid and embelin decreased the levels of the proinflammatory cytokines interleukin 1 beta (IL-1β), TNF-α, and interleukin 6 (IL-6), whereas pinellic acid inhibited the production of prostaglandins, leukotrienes, and nitric oxide (NO) in cell cultures [45].

Ten phenolic compounds in *Prosthechea michuacana* have been recorded. Pérez-Gutiérrez et al. [49] isolated a phenanthrene from the chloroform extract (4,6,7-trihydroxy-2-methoxy-8-(methylbut-2-enyl) phenanthrene-1,1′-4′,6′,7′-trihydroxy-2′-methoxy-8′-(methylbut-2′-enyl) phenanthrene) and two stilbenzenes (α-α’-dihydro,3′,5′,2-trimethoxy-3-hydroxy-4-acetyl-4′-isopentenylstilbene and gigantol). These compounds exerted antioxidant and lipid antiperoxidation activities, which are important for the treatment of oxidative tissue damage caused by the generation of reactive oxygen species. An additional study by Pérez-Gutiérrez et al. [51] isolated four flavones (scutellarein 6-methyl ether; dihydroquercetin; apigenin-7-O-glucoside; and apigenin-7-neohesperidoside) and one flavonoid (apigenin-6-O-β-d-glucopyranosyl-3-O-α-l-rhamnopyranoside). Of these, the compounds scutellarein 6-methyl ether, apigenin-7-neohesperidoside, and apigenin-6-O-β-d-glucopyranosyl-3-O-α-l-rhamnopyranoside exerted moderate hepatoprotective activity in the model of carbon tetrachloride (CCl_4_). Dihydroquercetin has shown antioxidant and hepatoprotective activities and the inhibition of lipid peroxidation [51].

In *Scaphyglottis livida*, seven phenolic compounds have been identified, including four stilbenes (gigantol; 3,4′-dihydroxy-3′,4,5-trimethoxybibenzyl (DTB); Batatasin III; and 3,4-dihydroxy-5,5-dimethoxybibenzyl) and three phenanthrene derivatives (coelonin, 3,7-dihydroxy-2,4,8-trimethoxyphenanthrene, and 3,7-dihydroxy-2,4-dimethoxyphenanthrene) [17]. Coelonin showed anti-inflammatory activity by inhibiting the expressions of IL-1β, IL-6, and TNF-α [107]. Many phenolic compounds have been isolated and identified from the epiphytic orchid *Vanilla planifolia*. However, the pharmacological activities of many of these compounds remain to be determined. The pharmacological effects of some phenolic compounds are listed below. Shyamala et al. [87] reported that the compounds 4-hydroxy-3-methoxybenzyl alcohol and 4-hydroxybenzyl alcohol exhibited antioxidant activities of 65% and 45%, respectively, using the beta carotene–linoleate method, and 90% and 50%, respectively, using the DPPH method. In contrast, 4-hydroxy-3-methoxybenzaldehyde (vanillin) showed lower antioxidant activity compared to the compounds previously mentioned [87]. Vanillin showed antitumor and antimicrobial properties, including the inhibition of bacterial biofilms. Vanillin at 500 µg·mL^−1^ reduced the development of biofilms induced by Candida albicans [108]. Finally, Kim et al. [109] reported that 4-hydroxy-3-methoxybenzyl alcohol (vanillyl alcohol) showed neuroprotective, antioxidant, and antiapoptotic effects in a study of 1-methyl-4-phenylpyridinium neurotoxin-induced cytotoxicity in MN9D dopaminergic cells.

### 6.3. Other Compounds

The glycoprotein N-acetyl-D-galactosamine was isolated from the epiphytic orchid *Laelia autumnalis*. This lectin possesses the biological activity of agglutinating desalted human erythrocytes of the human blood groups A, O, and B [34]. There are no reports about the presence of alkaloids identified in the analyzed orchids.

## 7. Validation of the Scientific Names of Medicinal Orchids

In the interest of systematics, it is necessary to correct the assignment of improperly applied species names in the scientific literature, as imprecise naming could lead to the pharmacological application of a natural product based on the mistaken identification of a plant. The literature consulted for this review reports several names that are no longer current because these names turned out to be synonyms of other plant names. In some cases, these names were applied to other species not distributed in Mexico or corresponded to similar species.

Coatzontecoxochitl was identified as *Stanhopea tigrina* Bateman, but Kunth recognized that Hernandez’s drawing corresponds to a non-described species and named this plant species *Anguloa hernandezii*, later changed to *Stanhopea hernandezii* Schltr. Tzauhtli was identified as *Bletia campanulata* Lex, but Hernández’s illustration corresponds to *Bletia jucunda* Linden & Rchb.f. Chichiltictepetzacuxochitl was named *Laelia autumnalis* (Lex.) Lindl., but Hernández’s illustration shows the characteristic inflorescence and flower of *Laelia speciosa*. Cozticcoatzontecoxochitl was identified as *Cattleya citrina* Mart. (=*Prosthechea citrina* (Lex.) W.E.Higgins). Francisco Hernández did not illustrate this plant, but its description, etymology (yellow flower with snake-head shape), and use (“flowers usually adorn crowns, garlands, and bouquets whose use is frequent and constant among the Indians”) suggest that it is *P. karwinskii*, which still has these uses in indigenous communities in Oaxaca [45]. Atzauhtli and acaltzauhtli were determined as *Cranichis speciosa* Lex. and *Cranichis tubularis* Lex., respectively [9,70], but these binomials are considered unplaced names and are therefore not accepted (https://powo.science.kew.org, accessed on 2 July 2024).

Hernández-Romero et al. [27] identified the species studied as *Epidendrum rigidum*, but this name corresponds to a species the presence of which has not been confirmed in Mexico; possibly the orchid evaluated should be referred to as *Epidendrum cardiophorum*. The correct name for the species evaluated by Estrada et al. [15,53] and Déciga-Campos et al. [17] must be *Scaphyglottis fasciculata* instead of *Scaphyglottis lívida*. *Oncidium cebolleta* (=*Trichocentrum cebolleta*) was the name assigned to the orchid reported to be a substitute for peyote used by Raramuri in northeastern Mexico [58,59,60,61] and evaluated by Pérez-Barrón et al. [85] in Morelos. However, this name corresponds to a species that, in Mexico, is distributed only in the Yucatan Peninsula, whereas in the states of Chihuahua and Morelos, this orchid is distributed as “rat tail” *Trichocentrum brachyphyllum*. *Specklinia gobryi* (Bateman ex Lindl.) F. Barros is a South American orchid, but it was reported by Alayón-Gamboa [20] as medicinal in Campeche, Mexico. Very similar species to this orchid, such as *S. marginata* Lindl. and *Pleurothallis choconiana* S. Watson, are distributed in the Yucatan Peninsula (southern Mexico).

Other issues for validating the taxonomic identities of the species detected in this review relate to the origin of the plant material for the evaluations, the reliability of the voucher specimen deposited in an herbarium collection, or the absence of reporting it in the publication. It is required, without exception, that all reports of medicinal orchids include a specimen deposited in an herbarium collection, the label of which contains information about the community where the medicinal use was recorded, the conditions (symptoms) for which the plant is used, and the plant parts employed. Studies on pharmacological evaluation must mandatorily include information about the voucher specimen, which allows for the taxonomic validation of the species used. Additionally, it would be advisable for these studies to include a photograph of the evaluated plant, enabling its identity to be corroborated.

## 8. Perspectives

As mentioned previously, further ethnobotanical studies are necessary to obtain detailed information on the medicinal uses of Mexican orchids. In many cases, the ethnomedicinal information is not complete or lacks detailed information.

The current national legislation (NOM-059-SEMARNAT-2010) on ecological protection for flora and fauna in Mexico needs to be updated [4]. This list includes eleven plant species cited in this review: *Epidendrum cnemidophorum* Lindl. (not endemic to Mexico, threatened); *Laelia autumnalis* (Lex.) Lindl. (endemic, with special protection); *Laelia furfuracea* Lindl. (endemic, with special protection); *Laelia speciosa* (Kunth) Schltr. (endemic to Mexico, with special protection); *Mormodes maculata* var. *unicolor* (Hook.) L.O.Williams (endemic to Mexico, threatened); *Prosthechea citrina* (Lex.) Withner (endemic to Mexico, with special protection); *Prosthechea karwinskii* (Mart.) J.M.H.Shaw, *Prosthechea vitellina* (Lindl.) W.E.Higgins (not endemic to Mexico, with special protection); *Stanhopea oculata* (Lodd.) Lindl. (not endemic to Mexico, threatened); *Stanhopea tigrina* Bateman ex Lindl. (endemic to Mexico, threatened); and *Vanilla planifolia* Jacks. ex Andrews (not endemic to Mexico, with special protection). However, no recent information is available about the endangered status of Mexican orchids.

The preservation and propagation of Mexican orchids should be encouraged. Our work group has focused on the micropropagation of several orchids with medicinal potential and distribution in Mexico [55,110,111,112]. Biotechnological approaches (plant tissue culture and other techniques) can be a useful strategy for the preservation of these plant species. National legislation should be created to include more sites as protected natural areas to avoid the plunder of orchids [113].

No studies are available on the pharmacological effects of Mexican orchids in clinical trials. Studies on the molecular mechanisms by which orchid extracts and their active compounds exert their pharmacological activities are necessary. In some cases, a possible mechanism of using the inhibitors of signaling pathways has been described. However, it remains to be fully understood how the plant extracts and their active compounds exert their pharmacodynamic actions on their therapeutic targets. In addition, pharmacokinetic and toxicity studies to obtain information before planning clinical trials are needed. These studies can provide information on the safe use of orchids and their active compounds in chronic-term assays.

The isolation and identification of active compounds from Mexican orchids should be encouraged. Phytochemical studies are an opportunity to obtain more secondary metabolites from Mexican orchids. This review indicates that many compounds obtained from orchids show similar or higher activities to those of reference drugs. Therefore, orchids are a source of active compounds with future medicinal potential. Metabolomic studies will be useful to obtain information on how orchids produce their active compounds.

## 9. Conclusions

Ethnobotanical studies have led to the obtainment of information on the medicinal uses of Mexican orchids. However, less than one-quarter of the medicinal orchids in Mexico have studies on their phytochemistry and pharmacology. Many compounds with pharmacological effects have been identified and isolated from medicinal orchids. Nevertheless, the mechanisms of action remain to be studied. Multidisciplinary work is necessary to obtain information that validates the medicinal uses of these medicinal plants. No clinical trials have been performed with medicinal orchids. The ecological preservation and propagation of these plants should be encouraged among the scientific community. Further work should be carried out with orchids with antinociceptive and spasmolytic activities, which show promising effects.

## Figures and Tables

**Table 1:** Medicinal species of the Orchidaceae family in Mexico.

Species Name	Common Name	Plant Part Used	Preparation Method	Ethnopharmacological Uses	State, Region, or Ethnicity	References
*Arpophyllum spicatum* Lex.	Tzauhxilotl	Pseudobulbs and stem	Decoction	Gastrointestinal disorders (dysentery)	Nahuatl	[9,10,11,12]
*Bletia campanulata* Lex.	Tzacuxochitl	Corms	Infusion	Gastrointestinal disorders (dysentery)	Nahuatl	[12,13]
*Bletia coccinea* Lex.	Tonalxochitl	Corms	Infusion	Gastrointestinal disorders (dysentery)	Nahuatl	[9,10,11]
*Bletia purpurea* (Lam.) A.DC.	NM	Corms	Infusion; crushed	Gastrointestinal disorders (dysentery)	Nahuatl; Tzetzal (Chiapas)	[14]
*Bletia reflexa* Lindl.	Tzautli	Corms	Infusion	Gastrointestinal disorders (dysentery)	Nahuatl	[9,10,11]
*Calanthe calanthoides* (A.Rich. & Galeotti) Hamer & Garay	NM	Corms and flowers	Crushed or powdered	To cicatrize wounds or burns	NM	[11,12]
*Camaridium densum* (Lindl.) M.A.Blanco	NM	Pseudobulbs and leaves	Infusion	Gastrointestinal disorders (dysentery); treatment of painful complaints; relaxant agent.	Catemaco, Veracruz	[1,15,16,17]
*Catasetum integerrimum* Hook.	Chinela; Cola de pato; Chi’i tku’uk; Trompa de puerco; Xøj cuxp tso’nabie; Nabie	Pseudobulbs	Crushed; infusion	Diabetes; diarrhea; renal disorders; to cicatrize wounds or burns; inflamed blows; to treat “Nacidos” (small inflamed tumor or grain on the skin); viper bite; dermatological problems	Zapoteca (Oaxaca); Mayans (Quintana Roo and Tabasco); Nahuatl (San Luis Potosí)	[1,11,13,18,19,20,21,22]
*Catasetum maculatum* Kunth	Ch’it cuc	Flowers	Cooked and grounded	Sores and tumors	Mayans (Yucatan)	[12]
*Chysis laevis* Lindl.	NM	Pseudobulbs and roots	Crushed and boiled in alcohol or water	To decrease fever	NM	Personal communication
*Cyrtopodium macrobulbon* (Lex.) G.A.Romero & Carnevali	Caña or cañaveral	Pseudobulbs	Crushed, roasted, and infusion	To cicatrize wounds or burns; inflamed blows; back affectation; painful urinary ailments	Acapulco, Guerrero; Tlaxiaco, Oaxaca; Tamasopo, San Luis Potosí; Chiapas	[14,23]
*Deiregyne eriophora* (B.L.Rob. & Greenm.) Garay	Cecetzi; Margaretilla	NM	NM	Asthma	Tlaquilpa, Veracruz	[24]
*Dichaea muricata* (Sw.) Lindl.	NM	Whole plant	Wash prepared	Conjunctivitis	NM	[25]
*Dichaea neglecta* Schltr.	Chhitë xkudzu belhë; espinazo de culebra	Branches	NM	Scare or bite caused by snake and swing scare	Santiago Camotlán and Totontepec Villa de Morelos, Oaxaca	[26]
*Dichromanthus cinnabarinus* (Lex.) Garay	Cutzis (for all species of *Dichromanthus* genera)	Leaves	Crushed and mixed with monnina (*Monina* sp.) leaf	Cold	San Juan Chamula, Chiapas	[18]
*Epidendrum anisatum* Lex.	NM	Stems	Infusion	Gastrointestinal disorders (such as dysentery)	Purepechan	[1]
*Epidendrum rigidum* Jacq.	NM	Stems	Infusion	Hydration	Quintana Roo	[27]
*Epidendrum cnemidophorum* Lindl.	Ech’wamal	Leaves	Infusion	Digestive disorders that produce flatulence	Oxchuc, Chiapas	[28]
*Epidendrum radicans* Pav. ex Lindl.	Semperavil ak’; Kilon vet; Tz’emani’; Krus tz’emani; Muk’tikil	Flowers and leaves	Mixed with Brugmansia leaves; mixed with *Polygonum punctatum*; infusion; crushed	Dandruff and grains; cough; burns; whooping cough	San Juan Chamula, San Andrés Larraínzar and San Pablo Chalchihuitán, Chiapas	[18]
*Epidendrum chlorocorymbos* Schltr	Simpre viva	Leaves	Infusion	Cholesterol lowering; ear pain; grains; sleep stimulation	Atzalan, Veracruz	[29]
*Epidendrum verrucosum* Sw.	Jutz’ep	NM	Crushed	To treat “nacidos”	Oxchuc, Chiapas	[18]
*Habenaria floribunda* Lindl.	Clavo cochinillo	Leaves	Infusion	Vaginal hemorrhage	Soteapan, Veracruz	[29]
*Isochilus latibracteatus* A.Rich. & Galeotti	Arroz largo	Whole plant	NM	Abdominal colic and gastrointestinal disorders	Soteapan, Veracruz	[12,29]
*Isochilus major* Cham. & Schltdl.	NM	Leaves	Crushed	Inflamed blows	Ixtaczoquitlan, Veracruz	[12,29]
*Isochilus* sp.	NM	Stems	Infusion	Gastrointestinal disorders	Ancient Mexican	[1]
*Laelia anceps* Lindl.	Flor de San Miguel; Flor de Todos Santos; Vara de San Diego	Pseudobulbs	NM	To treat pain and inflammation	Coatepec, Veracruz; San Luis Potosí	[30,31,32]
*Laelia autumnalis* (Lex.) Lindl.	Camote de San Diego; Flor de las Animas; Flor de Encino; Flor de la Calavera; Flor de Todos Santos; Flor de Catarina; Lirio de San Francisco	Pseudobulbs, leaves and flowers	Infusion; crushed and diluted in alcohol	Circulatory disorders; cough; hypertension; respiratory disorders; to heal the waist in postpartum; gastrointestinal disorders (such as dysentery)	Zirahuen, Michoacán; Puente de Ixtla, Tepoztlan and Tetela del Volcán, Morelos	[1,12,18,33,34,35,36,37]
*Laelia furfuracea* Lindl.	Gihtsl; Ita ndeka morada; Lirio morado; Lirio de San Francisco; Monja morada	Flowers	Infusion	Cough	San Pedro y San Pablo Teposcolula, Oaxaca	[38]
*Laelia speciosa* (Kunth) Schltr.	Chichiltictepetzacuxochitl (ancient Nahuatl, red and sticky flower of the hill) Deanta; Flor de Corpus; For de mayo; Itzamahua	Pseudobulbs and flowers	Infusion	Cough and inflamed blows	Valle del Mezquital, Hidalgo	[18,32]
*Malaxis* sp.	Jengibre	Corms	Infusion	Gastrointestinal disorders (such as dysentery)	Tepehuanes (Chihuahua)	[1]
*Maxillariella tenuifolia* (Lindl.) M.A.Blanco & Carnevali	Kowa nokcha	Roots	Crushed	Gastrointestinal disorders (such as dysentery)	Hueyapan de Ocampo and Soteapan, Veracruz	[39,40]
*Mormodes maculata* var. *unicolor* (Hook.) L.O.Williams	Flor de mayo	Pseudobulbs	Crushed	Inflammation caused by sprain dislocations	Zongolica, Veracruz	[29]
*Myrmecophila christinae* Carnevali & Gómez-Juárez	Flor de confesionario; cuerno; Homikim; Xonikni; X-yonixin	Pseudobulbs	Infusion	Pregnancy pain and wounds	Mayans in Calakmul, Campeche	[12,20]
*Myrmecophila tibicinis* (Bateman ex Lindl.) Rolfe	Hom-ikim	Pseudobulbs	Infusion or juice	Pregnancy pain to facilitate childbirth	Mayans (Yucatan)	[12,14,18,41]
*Nidema boothii* (Lindl.) Schltr.	NM	Whole plant	NM	Not used as a traditional or alternative remedy	Catemaco, Veracruz	[27]
*Oestlundia luteorosea* (A.Rich. & Galeotti) W.E.Higgins	Topixcamohtli	Pseudobulbs	Crushed in alcohol	Inflamed blows and headache	Zongolica, Veracruz	[29]
*Oncidium graminifolium* (Lindl.) Lindl.	Näjx pïj or tsäj pïj	Pseudobulbs	Infusion	Renal disorders	Santa Maria Tlahuitoltepec, Oaxaca	[42]
*Oncidium sphacelatum* Lindl.	Flor de mayo; Chorizo con huevo; Anis nikte’	Leaves	Warmed	Pain	Misantla, Veracruz	[18]
*Ornithocephalus inflexus* Lindl.	Puuts’mukuy	Sap	Spread	To treat insect bites	Mayans in Calakmul, Campeche	[20]
*Pleurothallis cardiothallis* Rchb.f.	Oo quia’ dsea ñu	Whole plant	Infusion; chewed	Gastrointestinal disorders; female sterility; to conceive male children	Zapotec (Oaxaca); Populaca (Veracruz)	[1]
*Ponera striata* Lindl.	NM	NM	NM	NM	Mayans in Calakmul, Campeche	[20]
*Prosthechea citrina* (Lex.) Withner	Cozticcoatzontecoxochitl	Pseudobulbs	Pseudobulbs cut in half used as poultice	Wound healing	Jesús del Monte and Ichaqueo, Michoacán	[12,14,37]
*Prosthechea karwinskii* (Mart.) Christenson	Ita ndeca amarilla; Lirio Amarillo; Monja amarilla	Pseudobulbs, leaves and flowers	Infusion; poultice	Diabetes; bleeding; to heal wounds or burns; cough; inflamed blows; to prevent risk of abortion	Tlaxiaco and Villa Sola de Vega, Oaxaca	[9,10,22,38,43,44,45]
*Prosthechea michuacana* (Lex.) W.E.Higgins	Aguanoso; Camote de agua; Näjx pïj o tsä jpïj	Pseudobulbs	Crushed; chewed; infusion	Diabetes; to cicatrize wounds or burns; circulatory disorders; hangover; inflamed blows; renal disorders	Ejutla de Crespo, Santa Catarina Ixtepeji, Santa Maria Tlahuitoltepec and Tlaxiaco, Oaxaca	[46,47,48,49,50,51,52]
*Prosthechea panthera* (Rchb.f.) W.E.Higgins	Jazmin	Roots	Two bundles of roots, boiled	Scabies	Amatenango del Valle, Chiapas	[18]
*Prosthechea pastoris* (Lex.) Espejo & López-Ferr.	Tzacutli	Pseudobulbs	Infusion	Gastrointestinal disorders (such as dysentery)	Nahuatl	[12,14]
*Prosthechea* sp.	Tzauhtli	Pseudobulbs	Infusion; chewed	Adhesive for poultices for bone fractures; bleeding; gastrointestinal disorders (such as diarrhea and dysentery)	Purepechan	[9,10]
*Prosthechea vitellina* (Lindl.) W.E.Higgins	Tzauxochitl	Pseudobulbs	NM	Gastrointestinal disorders (such as dysentery)	Nahuatl	[10]
*Rhyncholaelia digbyana* (Lindl.) Schltr.	Ch’it ku’uk, Piita, Xk’ubeenbaj nunup’le	Leaves	Crushed and placed in the wound	To cicatrize wounds and burns	Mayans in Calakmul, Campeche	[12,13,20,41]
*Rhynchostele bictoniensis* (Bateman) Soto Arenas & Salazar	Uch’al vo’; Camote de agua	Pseudobulbs and roots	Squeezed and mixed with salt: boiled	Baby cramps and headache	San Juan Chamula, Chiapas	[18]
*Scaphyglottis fasciculata* Hook.	Parasita menuda	NM	Infusion	Anti-inflammatory, antinociceptive, and relaxing activities	Catemaco, Veracruz	[29]
*Scaphyglottis livida* (Lindl.) Schltr.	Parasita	NM	Topically	To eliminate parasites, relieve stomachache, relax muscles; colic treatment; insect repellent; to prevent miscarriage	Los Tuxtlas (Veracruz)	[15,17,53]
*Sobralia macrantha* Lindl.	Atempanxochichocane; Cabolín; Lirio de San Antonio	Leaves	Crushed; liquated in water with lemon and honey added	Cough; fever; inflamed blows	Xocoyolo and Cuetzalan, Puebla; Chiconquiaco, Veracruz	[41,54]
*Specklinia* sp. (Bateman ex Lindl.) F.Barros	NM	NM	NA	NM	Mayans in Calakmul, Campeche	[20]
*Stanhopea hernandezii* (Kunth) Schltr.	Coatzontecomaxochitl: Cabeza de víbora	Flowers	Mixed with red corn and other herbs to make tortillas	Sunstroke and weakness	Ancient Mexican	[9,10,12,37]
*Stanhopea oculata* (Lodd.) Lindl.	Tehuanxochitl; Flor de la bestia	Pseudobulbs	Crushed and boiled	To reduce pain of women in labor	Zongolica, Veracruz	[29]
*Stanhopea tigrina* Bateman ex Lindl.	Cabeza de vibora; Toritos	Pseudobulbs, leaves, and roots	Powdered	To treat sunstroke, weakness, renal disorders, and mental disorders	Huasteca veracruzana (Veracruz); Huasteca Potosina (San Luis Potosí)	[12,55,56]
*Trichocentrum ascendens* (Lindl.) M.W.Chase & N.H.Williams	Cola de rata; cuerno de chivo; puknakché (Maya)	Whole plant	Crushed	Inflammation caused by splinter; “Limpia” (a ritual to prevent, diagnose, or cure a disease set); headaches; toothaches; stomachaches; kidney diseases	Soteapan, Veracruz; Yucatán; Quintana Roo	[19,29,57]
*Trichocentrum brachyphyllum* (Lindl.) R.Jiménez	Cola de iguana o cola de rata.	Leaves	Dried and chewed	To treat artery hypertension; as a hallucinogen; to treat abdominal colic	Raramuri (Chihuahua)	[12,58,59,60,61]
*Trichocentrum luridum* (Lindl.) M.W.Chase & N.H.Williams	Orejas de burro	Whole plant	NM	Treatment of heart conditions and asthma	Campeche, Chiapas, Oaxaca, San Luís Potosí, Tabasco, Veracruz, Yucatán	[12,62]
*Vanilla mexicana* Mill.	Vainilla sin perfume	Whole plant	Essential oil	Vermifuge	NM	[63]
*Vanilla planifolia* Jacks. ex Andrews	Tlilxochitl; Xanat; Vainilla	Fruits and flowers	Flowers squeezed in water; flowers powered and placed into a Magnolia flower; flavoring chocolate or drink water; mixed with mecaxochitl to provoke “regla” (menstruation); aromatherapy; infusion; decoction	Fatigue; gastrointestinal disorders; bleeding, diuretic; to treat nervous problems; to lower fever; to accelerate birth child; to attract dead fetuses; to provoke menstruation; “Mal aire” (a condition caused by the penetration of a harmful mist into the body); to facilitate digestion; skin tumors; dermatological problems; to treat hysteria, impotence, and rheumatism; to increase the energy of muscular systems	Misantla, Veracruz; Huasteca Potosina (San Luis Potosí); Puebla	[10,18,33]

NM: not mentioned.

## Data Availability

No new data were created or analyzed in this study. Data sharing is not applicable to this article.
